# Aldoxime dehydratases: production, immobilization, and use in multistep processes

**DOI:** 10.1007/s00253-024-13272-6

**Published:** 2024-11-15

**Authors:** Ludmila Martínková, Michael Kotik, Natalia Kulik, Barbora Křístková, Katarína Šťastná, Margit Winkler

**Affiliations:** 1 Laboratory of Biotransformation, Institute of Microbiology of the Czech Academy of Sciences, Vídeňská 1083, CZ-142 00 Prague, Czech Republic; 2 Laboratory of Photosynthesis, Centre Algatech, Institute of Microbiology of the Czech Academy of Sciences, Novohradská 237, 379 81 Třeboň, Czech Republic; 3 Faculty of Food and Biochemical Technology, University of Chemistry and Technology, Prague, Technická 5, 166 28 Prague, Czech Republic; 4 Department of Biochemistry, Faculty of Science, Charles University, Hlavova 2030/8, 128 44 Prague, Czech Republic; 5 Institute of Molecular Biotechnology, Graz University of Technology, Petersgasse 14, 8010 Graz, Austria; 6 Austrian Centre of Industrial Biotechnology GmbH, Krenngasse 37, 8010 Graz, Austria

**Keywords:** Aldoxime dehydratase, Nitrile synthesis, Biocatalyst, Multistep reaction, Immobilization

## Abstract

**Abstract:**

The synthesis of nitriles is of utmost importance for preparative organic chemistry. The classical routes are often associated with disadvantages such as toxicity of the reagents and drastic conditions. The uses of enzymes like aldoxime dehydratases (Oxds) and hydroxynitrile lyases constitute attractive benign alternatives. In this review, we summarize the recent trends regarding Oxds. Thousands of *oxd* genes were sequenced but less than thirty Oxds were investigated on protein level. We give an overview of these Oxds, their sequence analysis, conditions required for their overexpression, and their purification and assays. We then focus on the use of Oxds especially in multistep reactions combining the chemical or chemoenzymatic synthesis of aldoximes from different starting materials with the enzymatic dehydration of aldoximes to nitriles, possibly followed by the hydration of nitriles to amides. Progress in Oxd immobilization is also highlighted. Based on data published mainly in the last 5 years, we evaluate the industrial prospects of these enzyme processes in comparison with some other innovations in nitrile synthesis.

**Key points:**

• *Aldoxime dehydratases (Oxds) are promising for cyanide-free routes to nitriles*

• *A comprehensive overview of wet-lab explored Oxds is provided*

• *Recent trends include combining Oxds with other enzymes or chemical catalysts*

**Graphical Abstract:**

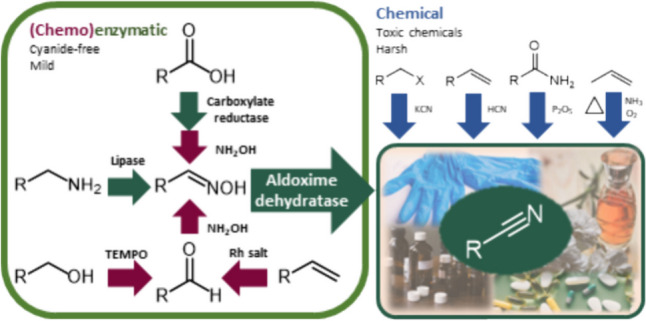

## Introduction

Nitriles are a highly sought-after group of compounds. They can be used as, e.g., solvents, fuels, fragrances or pharmaceuticals, and precursors in organic synthesis. They include bulk and fine chemicals (Betke et al. 2018). Some well-known examples of the former are the polymer precursors acrylonitrile and adiponitrile or acetonitrile and propionitrile which are widely used as solvents. Nitriles are also used as precursors of agrochemicals, drugs or surfactants (fatty amines from fatty nitriles) (Hinzmann et al. 2019b, 2020a, 2021; Yavuzer et al. 2023).

There is a number of pharmaceuticals that bear a cyano group (Gröger and Asano 2020; Chen et al. 2021). These are saxagliptin (Onglyza) and vildagliptin (Galvus) which serve for the treatment of type 2 diabetes mellitus, the antihistamine levocabastine, the calcium channel blocker verapamil, the anticonvulsant perampanel, the antiandrogens bicalutamide and enzalutamide, the anti-gout medicine febuxostat, and the antifungal isavuconazole. In veterinary medicine, trilostane (Vetoryl) is used for the therapy of hypercortisolism (Cushing’s disease) in dogs (Ramsey 2010).

**Fig. 1:**
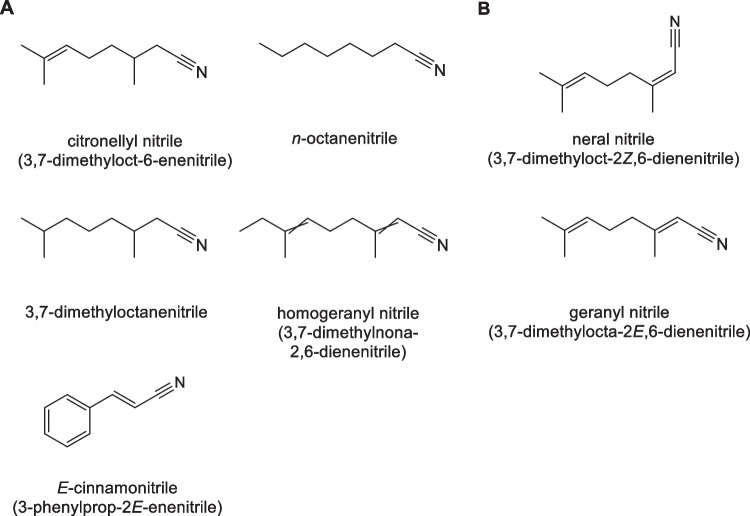
Examples of aroma nitriles. **A** Nitriles used as fragrances; **B** nitriles not recommended for use as fragrances due to health or environmental hazard

Several nitriles can be used as fragrances as they smell similar to the corresponding fragrance aldehydes and are often more stable. The use of some of them is considered safe, while others are associated with environmental or health hazards despite similar structures (Fig. 1). Thus, citronellyl nitrile (3,7-dimethyloct-6-enenitrile) and homogeranyl nitrile (3,7-dimethyl-2,6-nonadienenitrile) have been used in dozens of homecare products, *n*-octanenitrile and 3,7-dimethyloctanenitrile in cleansers, and *E*-cinnamonitrile (3-phenylprop-2*E*-enenitrile) in air fresheners and scented candles (Consumer Product Information Database, https://www.whatsinproducts.com). In contrast, the use of geranyl nitrile (dimethylocta-2*E*,6-dienenitrile, https://pubchem.ncbi.nlm.nih.gov/compound/1551246) or neral nitrile (3,7-dimethylocta-2*Z*,6-dienenitrile, https://pubchem.ncbi.nlm.nih.gov/compound/1551245) should be avoided due to chronic aquatic toxicity and genotoxicity hazards.

**Fig. 2:**
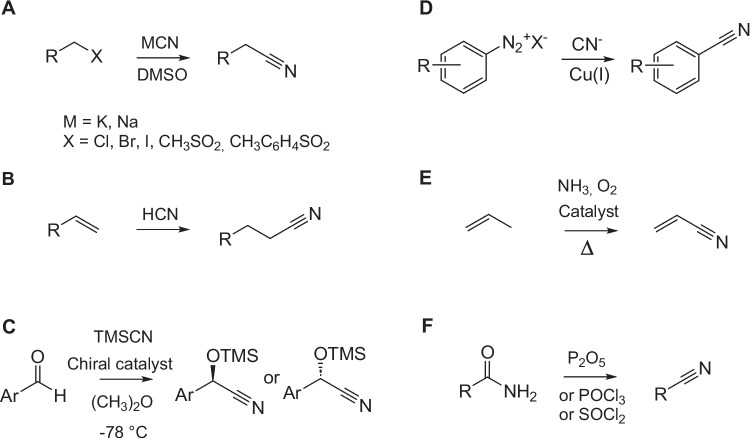
Classical routes to nitriles: **A** Kolbe nitrile synthesis in dimethyl sulfoxide (DMSO); **B** hydrocyanation (here: hydrocyanation of alkenes); **C** asymmetric cyanation with trimethylsilyl cyanide (TMSCN); **D** Sandmeyer cyanation; **E** ammoxidation (here: acrylonitrile synthesis); **F** amide dehydration

The classical methods (Fig. 2) used for nitrile synthesis are substitution of alkyl halides or other alkylating agents with simple cyanides (Kolbe nitrile synthesis), hydrocyanation of alkenes, ammoxidation of alkenes or aromatic hydrocarbons, dehydration of amides (Chen 2021; Hinzmann et al. 2021), or a Sandmeyer reaction of aromatic diazonium salts (Akhtar et al. 2022). Each of them suffers from certain disadvantages. For the substitution reaction (Fig. 2A) and the hydrocyanation (Fig. 2B), the highly toxic metal cyanides (KCN, NaCN) or HCN must be used in a stoichiometric ratio. An alternative cyanation agent, trimethylsilyl cyanide, which also allows for an assymetric cyanation (for example, see Fig. 2C) (Holmes and Kagan 2000a, b) has a similar toxicity. The Sandmeyer cyanation of a diazonium salt (Fig. 2D) with KCN or another CN^−^ donor and typically Cu(I) as catalyst (Akhtar et al. 2022) is limited to aromatic nitriles, and the diazonium salt is synthesized using the toxic nitrite. Ammoxidation (Fig. 2E) requires high temperatures and metal catalysts. Hazardous dehydrating agents (thionyl chloride, phosphoryl chloride, phosphorus pentoxide) or metal catalysts are needed for amide dehydration (Ganesan and Nagaraaj 2020) (Fig. 2F). The method choice depends on the type of the target nitrile. Thus, adiponitrile, acrylonitrile and fatty acid nitriles are preferentially produced by hydrocyanation, ammoxidation, and amide dehydration, respectively (Hinzmann et al. 2021).

Cyanide-free reactions that take place under mild conditions and do not require hazardous or expensive reagents or catalysts are highly desirable. For example, such reactions are catalyzed by enzymes that are involved in the synthesis of natural nitriles (Irmisch et al. 2014, 2015; Sørensen et al. 2018; Liu and Li 2024; Yamaguchi and Asano 2024). However, some of the enzymes such as β-cyano-l-alanine synthase (Kumano et al. 2016) or 7-cyano-7-deazaguanine synthetase (Winkler et al. 2015) have narrow substrate specificities (Yamaguchi and Asano 2024). In contrast, the scope of substrates transformed by hydroxynitrile lyases and aldoxime dehydratases (Oxds) is broad, which makes these enzymes interesting for nitrile synthesis.

The progress in the engineering and use of hydroxynitrile lyases has been reviewed recently (Priya and Padhi 2023). Another recent review addressed the nitrile-synthesizing enzymes and pathways as a whole with focus on their distribution in microorganisms, plants and animals, their natural functions, and the synthesis of volatile nitriles (Yamaguchi and Asano 2024). A recent study summarized the synthesis of nitriles in plants and insects (Yamaguchi 2024). Other reviews focused on Oxds, in particular their substrate specificity (Betke et al. 2018; Chen 2021), their in vivo functions (Rädisch et al. 2022) and their synthetic potential as a whole (Bhalla et al. 2018; Hinzmann et al. 2021) or emphasized on chiral nitriles (Gröger and Asano 2020; Domínguez de María 2021). The newest of these reviews were largely based on literature published until 2020. In addition, the most important features of nitrile biosynthesis by Oxds were summarized in a separate chapter in a recently published book on nitrile chemistry (Seth 2024).

In this study, we focus on recent advances in the application of Oxds, particularly in multistep (chemo)enzymatic reactions. First, we discuss the availability of Oxds and the production of catalysts based on them. We then summarize the recent uses of Oxds, while highlighting reaction parameters such as substrate concentrations, conversions, isolated yields, and space–time yields, which indicate the industrial prospects of the processes. Finally, we compare the processes catalyzed by Oxds with alternative reactions. The review is mainly based on the literature of the last five years.

## Aldoxime dehydratases: availability and catalyst forms

### Sequence diversity

The number of putative Oxds found by database searches depended on the database and software used. Thus, the Oxds were about 3000 according to BLAST searches of GenBank and UNIPROT (Křístková et al. 2023) and about 8000 according to 3DM software searches of the 3DM database from Bio-Prodict (Hinzmann et al. 2023a, b). The 3DM method is based on aligning three-dimensional protein structures unlike the common BLAST method based on aligning amino acid sequences. Only a small fraction of the putative Oxds was used for expression and subsequent characterization of the corresponding enzymes. The number of recombinantly expressed genes was 27, and enzyme activity was experimentally confirmed in 19 cases (Table 1). In addition, endogenous OxdSs was obtained from *Sclerotinia sclerotiorum* (Pedras et al. 2010)*.* So far, three X-ray structures of Oxds, originating from *Pseudomonas*, *Rhodococcus*, and *Bacillus* species, have been determined (Sawai et al. 2009; Nomura et al. 2013; Matsui et al. 2022). The wide occurrence of Oxds is not surprising as some aldoximes are precursors of plant defenses or defense compounds themselves, optionally complexed to glucosinolates or glycosides such as phenylacetaldoxime glucoside (Müller et al. 2024). The identification of Oxds acting on aromatic aldoximes has been challenging. Recently, however, OxdF1 from *Pseudomonas putida* F1 (Chen et al. 2021), OxdPsp from *Pseudomonas* sp. (Hinzmann et al. 2023b), and the M29G and F306A variants of OxdRE (Hinzmann et al. 2023a) proved to accept some of these substrates. OxdSs is exceptional in terms of substrate specificity. Out of 14 aldoximes tested, only indolyl-3-acetaldoxime, its analogs derived of propanal and butanal, and 4-hydroxy- and 4-methoxyphenylacetaldoxime (not phenylacetaldoxime) were shown to be substrates (Pedras et al. 2010).

**Table 1:** Aldoxime dehydratases investigated at protein level

Oxd	Origin	Acc. No	Length	Reference
OxdA	*Pseudomonas chlororaphis* B23	WP_024075760.1^b^	352	Nomura et al. 2013
OxdAA^a^	*Aggregatibacter actinomycetemcomitans *RhAA1	WP_005577064.1	234	Hinzmann et al. 2023b
OxdAsp	*Aspergillus ibericus* CBS 121593	XP_025572196.1	341	Pei et al. 2023
OxdB	*Bacillus *sp. OxB-1	BAA90461.1^c^	351	Matsui et al. 2022
OxdBr1	*Bradyrhizobium* sp*.* LTSPM299	WP_044589203.1	345	Rädisch et al. 2018
OxdBr2	*Bradyrhizobium* sp. WSM1253	WP_007594278.1	351	Křístková et al. 2023
OxdBT^a^	*Bacteroides thetaiotaomicron*	WP_008764895.1	131	Hinzmann et al. 2023b
OxdCp	*Corynebacterium pacaense* Marseille-P2417	WP_080796375.1	356	Winkler et al. 2023
OxdF1	*Pseudomonas putida* F1	ABQ78858.1	352	Chen et al. 2021
OxdFG	*Fusarium graminearum* MAFF 305135	BAE48794.1	363	Kato and Asano 2005
OxdFNn	*Fusobacterium nucleatum* ATCC 23726	WP_005902774.1	234	Hinzmann et al. 2023b
OxdFv	*Fusarium vanettenii* 77–13-4	XP_003042958.1	341	Křístková et al. 2023
OxdHsp	*Hydrogenophaga* sp. RAC07	WP_069048334.1	347	Hinzmann et al. 2023b
OxdHR	*Herbaspirillum rubrisubalbicans *M1	WP_058896488.1	349	Hinzmann et al. 2023b
OxdK	*Pseudomonas* sp. K-9	BAD98528.1	352	Kato and Asano 2006
OxdLC^a^	*Lactobacillus crispatus*	WP_060462053.1	220	Hinzmann et al. 2023b
OxdMR	*Methylobacillus rhizosphaerae*	WP_089375755.1	355	Hinzmann et al. 2023b
OxdPs^a^	*Pseudomonas syringae*	WP_060413740.1	129	Hinzmann et al. 2023b
OxdPsp	*Pseudomonas *sp. RIT-PI-q	WP_059405603.1	346	Hinzmann et al. 2023b
OxdRE	*Rhodococcus erythropolis*	BAD17969.1^d^	353	Sawai et al. 2009
OxdRG	*Rhodococcus globerulus*	BAC99076.1	353	Xie et al. 2003
OxdRYH3	*Rhodococcus* sp. YH3-3	WP_064442863.1	353	Kato et al. 1999
OxdSs	*Sclerotinia sclerotiorum*	XP_001597459.1	347	Pedras et al. 2010
OxdVP	*Variovorax paradoxus*	WP_047787064.1	353	Hinzmann et al. 2023b

^a^Activity detected for pUC18 expression but not pET28a expression

^b^pdb code 3W08

^c^pdb code 7F2Y, 72FZ, 7F30 (variant E85A)

^d^pdb code 3A15-3A18

Note: Hypothetical Oxds from *Streptomyces griseoruber*, *Fusobacterium nucleatum*, *Bacteroides thetaiotaomicron*, and *Parabacteroides goldsteinii* were expressed, but no activity was detected (Hinzmann et al. 2023b)

**Fig. 3:**
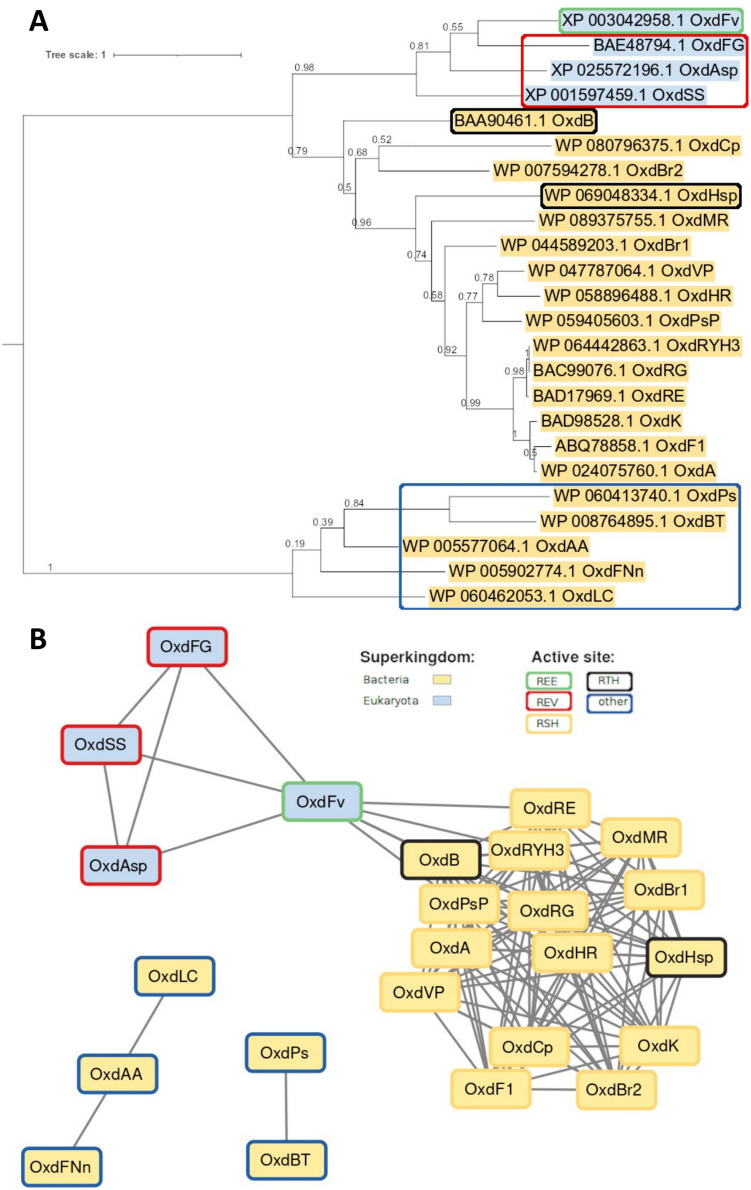
Sequence analysis of aldoxime dehydratases investigated at protein level. **A** Phylogenetic tree constructed using maximum likelihood method and w/freq. model (Jones et al. 1992) in MEGA X (Kumar et al. 2018). The proportion of trees in which the associated taxa clustered together during bootstrap evaluation is shown above the branches. The branch length in the tree is scaled according to the number of substitutions per site. All positions with less than 70% site coverage were eliminated leaving a total of 325 positions in the final dataset. **B** Sequence similarity network constructed using the online tool (EFI (Enzyme Similarity Tool), https://efi.igb.illinois.edu/efi-est/) with a threshold of 20% sequence identity in the local alignment for the display of connections (Zallot et al. 2019). Data are visualized with Cytoscape (Shannon et al. 2003). The color scheme is the same for A and B and is shown in B

Analysis of the evolutionary relationships between the characterized Oxds suggests that fungal and bacterial Oxd have rather different sequence properties, and, moreover, the fungal Oxds seem to have more diversity in active site residues. The characterized fungal Oxds are not only evolutionarily located in a different branch but have a large evolutionary distance from bacterial Oxds (Fig. 3A).

Analysis of the sequence similarity network allowed even better discrimination between Oxds with different active site residues. OxdLC, OxdAA, OxdFNn, OxdPs, and OxdBT (Hinzmann et al. 2023b) are split into separate clades and have less than 20% sequence identity to all other Oxds (missing connections in Fig. 3B). Remarkably, these proteins are significantly shorter (with 129 to 234 amino acid residues) than the “conventional” Oxds with typically about 350 amino acid residues (Table 2). Initial screens with enzymes expressed with a pUC18 vector indicated a certain activity also for the “short” Oxds. However, after subcloning the genes in pET28a, rescreening revealed no Oxd activity in these proteins, suggesting that the above results were false positives.

**Table 2:** Determination of aldoxime dehydratase substrates and products (examples)

Aldoxime	Nitrile	Method	Reference(s)
Aliphatic compounds			
Butyraldoxime	Butyronitrile	GC	Zheng et al. 2022; Hinzmann et al. 2023b
Valeraldoxime	Valeronitrile	GC	Křístková et al. 2023
Isovaleraldoxime	Isovaleronitrile	GC	Rädisch et al. 2018
*n*-Hexanaldoxime	*n*-Hexanenitrile	GC	Hinzmann et al. 2020a; Zheng et al. 2022
*n*-Heptanaldoxime	*n*-Heptanenitrile	GC	Yavuzer et al. 2023; Chen et al. 2021; Zheng et al. 2022
*n*-Octanaldoxime	*n*-Octanenitrile	GC	Hinzmann et al. 2020a,b; Hinzmann et al. 2023b; Yavuzer et al. 2023
*n*-Octanedialdoxime	*n*-Octanedinitrile	GC	Hinzmann et al. 2020a
*n*-Nonanaldoxime	*n*-Nonanenitrile	GC	Plass et al. 2019
*n*-Decanaldoxime	*n*-Decanenitrile	GC	Hinzmann et al. 2020a
*n*-Dodecanaldoxime	*n*-Dodecanenitrile	GC	Yavuzer et al. 2023
*n*-Tetradecanaldoxime	*n*-Tetradecanenitrile	GC	Yavuzer et al. 2023
Citronellal oxime	Citronellyl nitrile	GC	Zheng et al. 2022; Pei et al. 2023
Alicyclic compounds			
Cyclopentanecarbaldehyde oxime	Cyclopentanecarbonitrile	GC	Hinzmann et al. 2023b
Cyclohexanecarbaldehyde oxime	Cyclohexanecarbonitrile	GC	Hinzmann et al. 2023b
Arylaliphatic compounds			
Phenylacetaldoxime	Phenylacetonitrile	RP-HPLC, GC	Zheng et al. 2022; Hinzmann et al. 2023b
2-Phenylpropionaldoxime	2-Phenylpropionitrile	RP-HPLC	Rädisch et al. 2018; Chen et al. 2024
3-Phenylpropionaldoxime	3-Phenylpropionitrile	RP-HPLC, NP-SFC, GC	Křístková et al. 2023; Hinzmann et al. 2023b
*E*-Cinnamaldoxime	*E*-Cinnamonitrile	RP-HPLC	Křístková et al. 2023; Pei et al. 2023
Aromatic compounds			
Benzaldoxime, substituted benzaldoximes	Benzonitrile, substituted benzonitriles	RP-HPLC	Zheng et al. 2022; Xiao et al. 2023
Vanillinoxime	Vanillonitrile	RP-HPLC	Winkler et al. 2023
Heterocyclic compounds			
2-Furfuraldehyde oxime	2-Furonitrile	RP-HPLC, GC, UV-spectrometry	Choi et al. 2020; Zheng et al. 2022
3-Methyl-2-thiophene-carbaldehyde oxime	3-Methyl-2-thiophene-carbonitrile	RP-HPLC	Zheng et al. 2022
*E*-Pyridine-3-carbaldehyde oxime	3-Cyanopyridine	GC, UV-spectrometry	Choi et al. 2020
4,5-Dihydroisoxazoles			
Benzisoxazoles	Cyanophenoxides	UV-spectrometry	Miao et al. 2017
5-Phenyl-4,5-dihydroisoxazole and analogs	3-Hydroxynitriles	Chiral HPLC, chiral GC	Zheng and Asano 2020; Zheng and Asano 2021

*RP* reversed-phase, *NP-SFC* normal-phase supercritical fluid chromatography

This sequence analysis reveals a lack of information on a relatively large group of Oxds, including those with REV or REE active sites (Fig. 3B), which are widespread in the library of fungal homologs (Křístková et al. 2023). Further experiments will be required to elucidate the relationships between active site residues and substrate specificities in different Oxd clades.

### Overproduction and purification

To the best of our knowledge, all recombinant Oxds, either of bacterial or fungal origin, have been overproduced in *Escherichia coli* hosts. The heterologous expression of *oxd* genes has often been found challenging. In this respect, low expression levels and inclusion body formation of the target Oxd protein were reported (Choi et al. 2019; Hinzmann et al. 2023b). In order to alleviate these problems, special cloning, cultivation and induction strategies have been devised, such as host-targeted codon optimization of the *oxd* genes (Hinzmann et al. 2023b; Křístková et al. 2023; Pei et al. 2023), elimination of codon bias using engineered *E. coli* expression hosts (e.g., BL21-CodonPlus(DE3)-RIL) (Oinuma et al. 2003; Kato and Asano 2006), low temperatures (15–25 °C) (Oinuma et al. 2003; Xie et al. 2003; Kato et al. 2006; Rädisch et al. 2018; Chen et al. 2021), extended cultivation periods of up to 7 days (at 15 °C; Oinuma et al. 2003), and the omission of an external inducer during the protein overproduction phase in connection with T7 promoters and lac operators (leaky expression) (Hinzmann et al. 2023b). Additional strategies to improve the production of active Oxds in recombinant *E. coli* strains have been reported: lower aeration levels brought about by cultivation in shaken tubes with high culture broth volumes resulting in lower growth rates (Kato et al. 2004; Kato and Asano 2005) and optimization of the position of the His_6_-tag (Kato and Asano 2006). These are all common strategies for optimized heterologous protein production (Rong et al. 2023). The use of a 5-L reactor with a 3-L working volume enabled to efficiently control the level of dissolved oxygen during the production of whole cells carrying both OxdF1 and nitrile hydratase (NHase) (Zheng et al. 2022). Although Oxds are heme-containing enzymes, their heterologous expression as active enzymes did not so far make use of special *E. coli* strains engineered for this purpose (Fiege and Frankenberg-Dinkel 2020).

Purification strategies for Oxds have followed the general strategy of using affinity tags attached to recombinant proteins for facilitating protein purification (Mishra 2020). Most popular have been His_6_-tags in conjunction with immobilized metal ion affinity chromatography using Co^2+^ or Ni^2+^ ion-containing resins. Occasionally, a sequence of different chromatographic separation steps with conventional purification media has been used for purifications of both recombinant and non-recombinant Oxds (Oinuma et al. 2003; Xie et al. 2003; Pedras et al. 2010; Nomura et al. 2013). It is noteworthy that partial heme loss has been observed during the purification of some Oxds (Kato et al. 2000; Oinuma et al. 2003; Xie et al. 2003). It is also worth mentioning that many Oxds have been found only barely thermostable (Kato et al. 2004; Křístková et al. 2023; Pei et al. 2023). In this regard, several authors reported instability issues with Oxds (Hinzmann et al. 2020a, 2023b), which was one of the reasons for preferring whole cells to purified enzymes as catalysts in biotransformations (see below).

### Reaction conditions

In all Oxds investigated so far, a heme B prosthetic group appears to be the key reaction center, where the aldoxime functionality gets in close contact to the heme iron. According to the proposed reaction mechanisms (Chen et al. 2021; Pei et al. 2023), the heme iron in its ferrous state is essential for the first step of the catalytic reaction. This explains why Oxd activities were often shown to be enhanced under anaerobic and/or reducing conditions. For instance, a marked increase in activity was reported for OxdRG (Xie et al. 2003), OxdF1 (Chen et al. 2021), and OxdA (Zheng and Asano 2020) in the presence of Na_2_S_2_O_4_, while a similar effect was observed with Na_2_S in OxdRE (Zheng and Asano 2020). Another example is OxdSs with a dramatic increase in its activity in the presence of Na_2_S_2_O_4_ under anaerobic conditions compared with aerobic conditions (Pedras et al. 2010). The addition of iron salts in combination with reducing agents was also reported to have positive effects on Oxd activities (Kato et al. 2004; Křístková et al. 2023). These effects must be taken into account in activity assays of purified Oxds, whereas the activities of whole cells can usually be determined in suitable buffers without additives and under aerobic conditions.

### Determination of substrates and products

To monitor the reactions, the concentrations of aldoximes and nitriles were usually determined by HPLC or GC (Table 2). Aliphatic and alicyclic aldoximes and nitriles were generally determined by GC, and GC was also used for some of the arylaliphatic and heterocyclic compounds. Reversed-phase HPLC was suitable to determine most of the arylaliphatic, aromatic, and heterocyclic aldoximes and nitriles. A spectrophotometric method was developed for the determination of the conversion of 2-furfurylaldehyde oxime and *E*-pyridine-3-carbaldehyde oxime to nitriles (Choi et al. 2020) or benzisoxazoles to nitriles (Miao et al. 2017). Similar methods based on the difference in the spectra of substrate and product can accelerate the monitoring of Oxd-catalyzed reactions.

### Catalyst forms

Oxd catalyzed reactions were largely performed with cell suspensions, but immobilized Oxds began to be investigated recently. The first of them were prepared based on whole *E. coli* cells. For example, *E. coli* carrying OxdB from *Bacillus* sp. OxB-1 was immobilized on a highly hydrophilic acrylic acid polymer. The resulting catalyst was functional in cyclohexane (a solvent with a high log P), with an over 99% conversion of 0.5 M *n*-octanaldoxime (82% isolated yield). The results obtained with other solvents with lower log *P* values (methyl-*tert*-butyl ether, toluene, dichloromethane) were inferior. The preference of high log *P* (highly apolar) solvents in biocatalysis is well-known. The rationale behind this effect is the low tendency of the apolar solvents to strip or “dry-out” (Hinzmann et al. 2019a) water from the enzyme catalyst. The same catalyst was also used to produce a 1-mL packed bed column, and the conversion of 0.1 M *n*-octanaldoxime was maintained over 95% for 3 h in cyclohexane in flow mode.

However, this catalyst is not appropriate for aqueous media, as this would destabilize the binding of the cells on the support. Therefore, biocatalysts suitable for aqueous environments were produced by immobilizing the cells in calcium alginate. The catalyst was optionally coated with tetraethyl orthosilicate to increase its hydrophobicity, which was important for the affinity of the substrate to the carrier. This immobilization increased the stability of OxdB whole-cell catalyst in 10% ethanol, and the immobilizate was used three times for the dehydration of 100 mM *n*-octanaldoxime with 70–88% conversion (Hinzmann et al. 2020b).

Other immobilized Oxds were prepared from purified enzymes. The enzymes were bound on the supports by hydrophobic, covalent (Hinzmann et al. 2020b), and affinity interactions (Křístková et al. 2024). Almost all the protein applied was bound on the supports (Hinzmann et al. 2020b), but the residual activity was found to be insufficient. OxdRE retained 15–20% activity and OxdB 10% activity on a hydrophobic or an amino (glutaraldehyde-activated) support. The stability of the immobilizates in the presence of acetonitrile, methanol, or dimethyl sulfoxide (20% each) was also unsatisfactory, the activity already decreasing after 15-30 min (Hinzmann et al. 2020b), which suggested to focus on whole-cell immobilizates (see above). In another study, OxdFv and OxdBr1 were bound to Ni–NTA through their *N*-terminal His_6_-tags. The residual activity was not determined, but a complete conversion of 5–15 mM phenylacetaldoxime or 5 mM cinnamaldoxime was achieved with the immobilizates, which were also recyclable (at least 22 times for OxdBr1). Moreover, this immobilization method did not require a prior purification but could also be carried out with cell-free extracts, while immobilization was combined with partial purification (Křístková et al. 2024). Similarly, immobilized OxdPsp was prepared by combining protein separation using the aqueous two-phase system (ATPS) with enzyme adsorption on a macroporous resin. The immobilizate was used for the enantioselective dehydration of (*E*)-2-phenylpropionaldoxime to the corresponding *S*-nitrile with 94% e.e. and was at least three times recyclable (Chen et al. 2024). In this and similar reactions of chiral aldoximes (Gröger and Asano 2020; Domínguez de María 2021), the *E*- vs. *Z*-configuration of the substrate is decisive for enantioselectivity. The hypothesis that the size of the cavity in the active site of Oxds plays a key role was confirmed by the study of mutants with reduced cavity size and improved enantioselectivity (Yavuzer et al. 2021).

## Cascade reactions

**Fig. 4:**
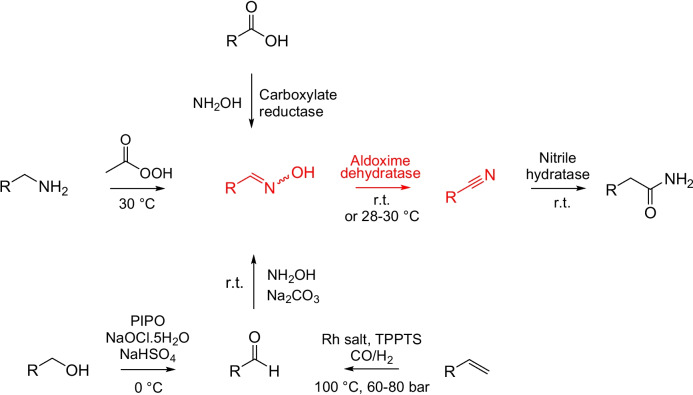
Multistep reactions of diverse precursors to nitriles. Aldoxime dehydratase is used in the oxime-to-nitrile step (Plass et al. 2019; Hinzmann et al. 2020a; Zheng et al. 2022; Winkler et al. 2023; Horvat et al. 2022; Xiao et al. 2023; Terhorst et al. 2020) (in red). Nitriles can be directly converted to amides (Zheng et al. 2022). PIPO, polymer-immobilized TEMPO (2,2,6,6-tetramethylpiperidinyloxy); TPPTS, (tris(3-sulfophenyl)phosphine trisodium salt); r.t., room temperature

Oxds were integrated in several multistep reactions, where the aldoximes were obtained from alkenes (Plass et al. 2019; Terhorst et al. 2020), alcohols (Hinzmann et al. 2020a), aldehydes (Zheng et al. 2022), carboxylic acids (Horvat et al. 2022; Winkler et al. 2023), or amines (Xiao et al. 2023) (Fig. 4).

### Synthesis of nitriles and amides from aldehydes

The synthesis of nitriles from aldehydes via oximes was developed early during the investigation of Oxds. The oximes prepared by condensation of aldehydes and hydroxylamine were isolated and used for the next step catalyzed by whole cells of *Rhodococcus* sp. YH3-3, in which the NHase was inactivated with a combination of 5 mM of 2-mercaptoethanol and 1 mM of DTT at 40 °C (Kato et al. 1999) or *E. coli* harboring the *oxd* gene from *Bacillus* sp. OxB-1 (Xie et al. 2001). Phenylacetonitrile was produced from 500 mM substrate (aldoxime) with 89% isolated yield, 3-phenylpropionitrile from 750 mM substrate with 90% isolated yield, and several aliphatic C_4_-C_6_ nitriles from 100 to 300 mM substrate concentration with up to 100% conversion and 46–56% isolated yields, within 2–20 h (Xie et al. 2001). Thus, the space-time yields (STYs) reached up to 10 g/L/h. In addition, 3-cyanopyridine and 2-furonitrile were prepared from 50 and 100 mM substrates with 98% and 62% isolated yields within 105 and 75 min, which corresponds to 2.92 and 4.62 g/L/h STY, respectively (Kato et al. 1999). A single point mutation (N266S) in OxdRYH3 increased the enzyme‘s potential for the production of 2-furonitrile increasing its specific activity for 50–100 mM substrate several times (Choi et al. 2020).

**Fig. 5:**
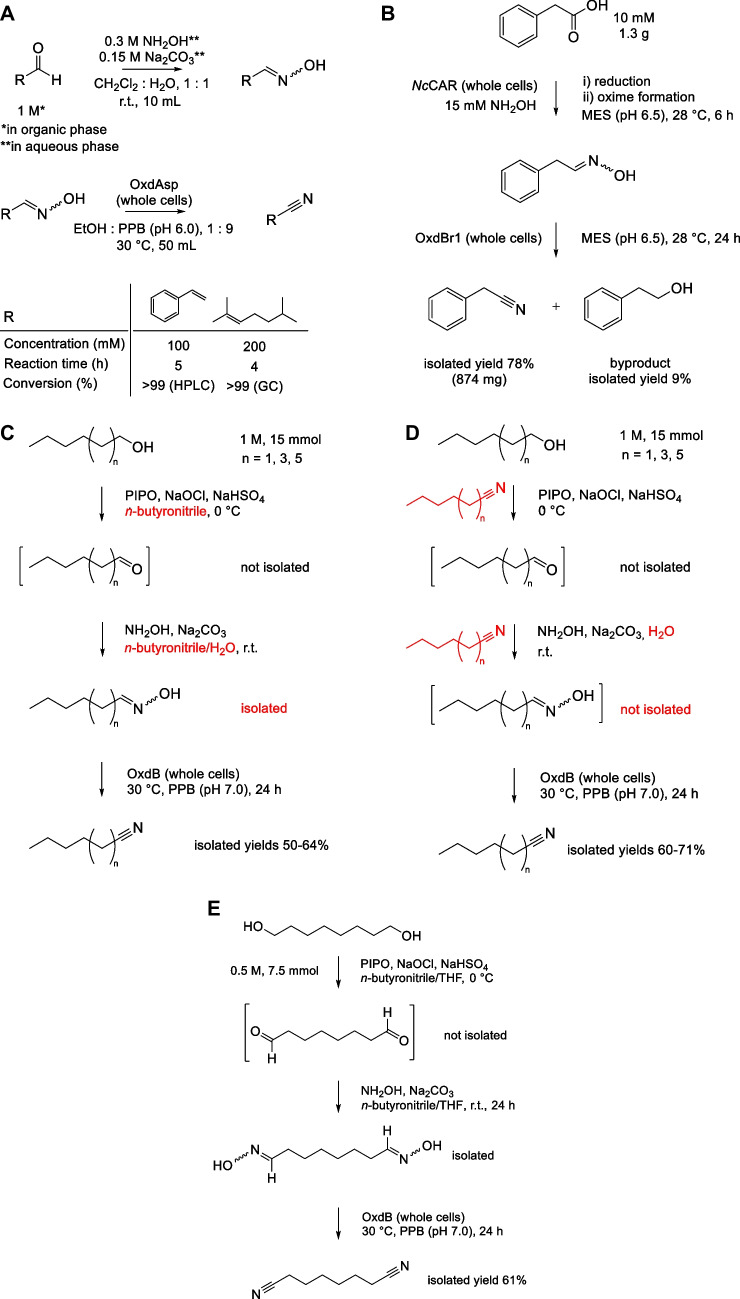

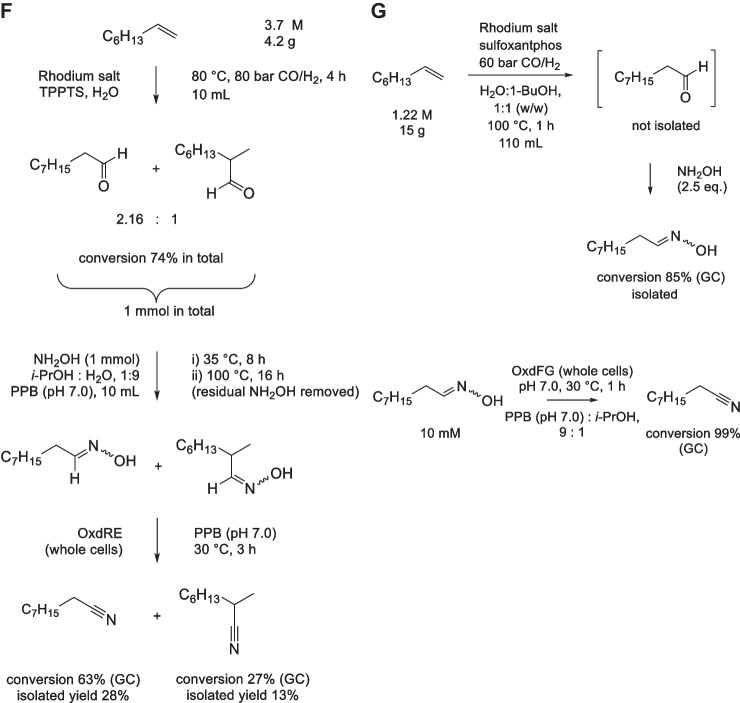
Chemoenzymatic syntheses of (aryl)aliphatic nitriles from **A** aldehydes, **B** carboxylic acids, **C**,** D** alcohols, **E** dialcohols, and **F**,** G** alkenes. **A** *E*-3-Phenylprop-2-enal (cinnamaldehyde) and 3,7-dimethyloct-6-enal (citronellal) were transformed to aldoximes with hydroxylamine and isolated. *E*,*Z*-Cinnamaldoxime and *E*,*Z*-citronellal oxime thus obtained were transformed to nitriles with whole cells (30 mg wet cells/mL) of *Escherichia coli* carrying the enzyme OxdAsp from *Aspergillus ibericus* (Pei et al. 2023). **B** Phenylacetic acid was reduced by carboxylate reductase *Nc*CAR from *Neurospora crassa* (*E. coli* cells, ≈6 mg dry cells/mL), and the crude product was then transformed by aldoxime dehydratase OxdBr1 from *Bradyrhizobium* sp. (*E. coli* cells, ≈3 mg dry cells/mL) (Winkler et al. 2023). **D–F** Aliphatic mononitriles were synthesized from the corresponding alcohols (**C**) with or (**D**) without intermediate (aldoxime) isolation (differences highlighted in red). **E** An analogous route to *n*-octanedinitrile was performed with aldoxime isolation. The aldoximes were transformed by aldoxime dehydratase OxdB from *Bacillus* sp. OxB-1. The catalyst was *E. coli* wet whole cells in free form (33 mg wet cell/mL) or immobilized form (Hinzmann et al. 2020a). The latter was based on whole cells adsorbed to an acrylic acid resin according to a previous study (Hinzmann et al. 2019a). **F** *n*-Nonanal and 2-methyloctanal were prepared by hydroformylation. An aliquot of the product (1 mmol) was taken for condensation with hydroxylamine followed by enzymatic dehydration with aldoxime dehydratase OxdRE from *Rhodococcus erythropolis* (*E. coli* cells, 50 mg wet weight/mL) (Plass et al. 2019). **G** Nonanal oxime was prepared by hydroformylation and aldoxime formation in “one pot.” The product was dehydrated using aldoxime dehydratase OxdFG from *Fusarium graminearum* (*E. coli* cells, 50 mg wet weight/mL) (Terhorst et al. 2020). PPB, potassium phosphate buffer; MES, 2-(*N*-morpholino)ethanesulfonic acid buffer; PIPO, polymer-immobilized TEMPO (2,2,6,6-tetramethylpiperidinyloxy); r.t., room temperature; TPPTS, (tris(3-sulfophenyl)phosphine trisodium salt)

Recently, the synthesis of cinnamonitrile and citronellyl nitrile from the corresponding aldehydes was demonstrated using a new Oxd from *Aspergillus ibericus* (enzyme OxdAsp) (Pei et al. 2023). Both aldoxime synthesis and dehydration proceeded under mild conditions, with high concentrations of substrates (1 M and 100–200 mM, respectively). The aldoxime to nitrile reactions proceeded with an almost full conversion (Fig. 5A), providing 2.58 g/L/h and 7.56 g/L/h STY for cinnamonitrile and citronellyl nitrile, respectively.

**Fig. 6:**
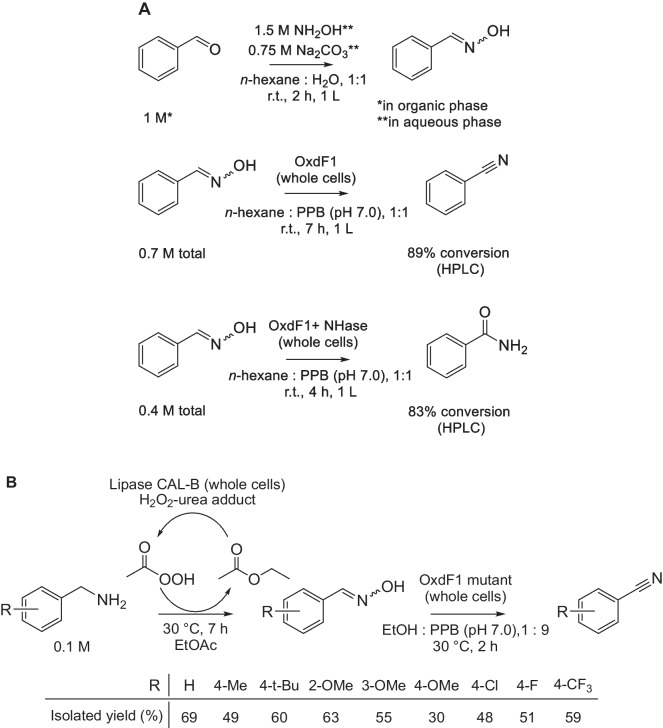
Chemoenzymatic syntheses of aromatic nitriles from **A** benzaldehyde and **B** benzylamines. **A** Benzaldehyde was transformed to benzaldoxime with hydroxylamine, and the organic phase containing the product was directly used for the transformations to benzonitrile with *E. coli* whole cells (30 g dry cells/L) carrying aldoxime dehydratase OxdF1, or to benzamide with *E. coli* whole cells (30 g dry cells/L) carrying OxdF1 and nitrile hydratase (NHase). The enzymatic reactions were carried out in fed-batch mode (Zheng et al. 2022). **B** Ethylacetate (solvent) was transformed by lipase B from *Candida antarctica* (CAL-B; *E. coli* lyophilized cells, 6 mg/mL) to peracetic acid which reacted with benzylamines to afford benzaldoximes. These were transformed to benzonitriles by aldoxime dehydratase from *Pseudomonas putida* F1—mutant OxdF1 L318F/F306Y (*E. coli* cells, 33 mg wet weight/mL). The nitrile yield was calculated per benzylamine (Xiao et al. 2023). PPB, potassium phosphate buffer; r.t., room temperature

Nitriles were also prepared from a variety of aromatic and aliphatic aldehydes without purifying the oximes (Zheng et al. 2022). OxdF1 was suitable to dehydrate all the oximes. Optionally, the nitriles were then hydrated to amides using a NHase from *Aurantimonas manganoxydans*. The amides were largely obtained with satisfactory (ca. 40–70%) isolated yields. In addition, the syntheses of benzonitrile and benzamide from benzaldoxime were carried out on a 1-L scale with STYs of 9-10 g/L/h (Fig. 6A).

### Synthesis of nitriles from carboxylic acids

Carboxylic acids are attractive substrates due to their availability from sustainable resources and stability in comparison to reactive aldehydes. To obtain aldoximes, they were reduced by carboxylate reductase (CAR), and the resulting aldehydes were reacted in situ with hydroxylamine. CARs accept a broad variety of substrates (Winkler and Ling 2022), including (aryl)aliphatic and aromatic substrates (Horvat and Winkler 2020), but not substrates with substitutions in vicinity of the carboxylic acid group. Oxds are highly efficient in dehydration of (aryl)aliphatic compounds, but their ability to transform aromatic aldoximes is limited. Therefore, the choice of a compatible CAR and Oxd for a certain reaction can be challenging.

A proof of concept was obtained for the synthesis of *n*-hexanenitrile (capronitrile) and phenylacetonitrile, on an analytical scale (Horvat et al. 2022). Several potential substrates were then tested with panels of CARs and Oxds on an analytical scale. Suitable CAR-Oxd combinations were found for most of the carboxylic acids investigated (butyric, valeric, caproic, benzoic, phenylacetic, and 3-phenylpropionic acid). The conversions (determined by HPLC or GC) varied from 80 to >99%. However, an over-reduction of some carboxylic acids to alcohols was observed. On a preparative scale, a sequential one-pot cascade reaction was carried out for the synthesis of phenylacetonitrile (874 mg) with an isolated yield of 78% (Winkler et al. 2023) (Fig. 5B). The by-product alcohol was also found in this case.

### Synthesis of nitriles from alcohols

The route from renewable carboxylic acids to nitriles can also occur via alcohols (Hinzmann et al. 2020a) that are obtained from the acids, e.g. by catalytic hydrogenation. A multistep process from alcohols to nitriles was proposed that involved oxidation of the alcohol to the aldehyde with a polymer-immobilized TEMPO catalyst (PIPO), condensation of the aldehyde with hydroxylamine, and enzymatic dehydration of the resulting aldoxime. Proof of concept was established for *n*-hexanenitrile, *n*-octanenitrile, and* n*-decanenitrile, which were produced in 50-64% overall yield, while the aldoxime intermediates were isolated (Fig. 5C). The process was then optimized to be run without intermediate isolation (Fig. 5D). Thus, the organic phase from the synthesis of aldoxime was directly used for the enzymatic step, which could be performed not only with the immobilized Oxd catalyst which is resistant to organic solvent (see above) but also with a whole-cell suspension, provided that the target nitrile was also used as solvent. Analogously, *n*-octanedinitrile (suberonitrile) was synthesized (Fig. 5E), but the route without aldoxime isolation was not possible in this case: the use of *n*-octanedinitrile as solvent resulted in precipitation of the oxime intermediate (Hinzmann et al. 2020a).

### Synthesis of nitriles from alkenes

The first step in the three-step process from alkene to nitrile is hydroformylation, which is well developed, but the drawback of which is the formation of isomers (Fig. 5F). The next two steps are the same as in the above multistep processes, i.e., condensation of the aldehyde with hydroxylamine followed by the enzymatic dehydration of aldoxime (Fig. 5F). The multistep reaction was demonstrated for 1-octene with *n*-nonanenitrile as the final product in a 28% overall yield and *iso*-nonanenitrile as a side product in a 13% overall yield (Plass et al. 2019).

Also one-pot formation of aldoxime from an alkene with subsequent synthesis of nitrile was reported (Terhorst et al. 2020). The optimization of reaction conditions enabled to combine the hydroformylation step with the aldehyde–hydroxylamine condensation step without isolating the aldehyde. The process was primarily demonstrated for the synthesis of *n*-nonanenitrile obtained with an overall yield of 85% (Fig. 5G) and a 95% selectivity for the targeted (linear) isomer. In addition, the use of the same reaction sequence for the synthesis of other (aliphatic, arylaliphatic) nitriles was also proposed, using various Oxds (Terhorst et al. 2020).

### Synthesis of nitriles from benzylamines

A route from amines to nitriles was shown for substituted benzylamines (Xiao et al. 2023). In the first step, the benzylamines were chemically oxidized to aldoximes with peracetic acid. This oxidant was produced from ethyl acetate in situ with a lipase catalyst, according to an earlier work (Méndez-Sánchez et al. 2017). At the same time, ethyl acetate served as the solvent. Amylacetate could be used analogously. The intermediate benzaldoxime was isolated by phase separation. The second step was carried out with a whole-cell catalyst based on a mutant of OxdF1 with improved kinetic behavior. Benzonitriles and substituted derivatives were obtained from 100 mM substrates largely in good isolated yields (Fig. 6B) and up to over 1 g/L/h STY.

### Synthesis of nitriles from dihydroisoxazoles

**Fig. 7:**
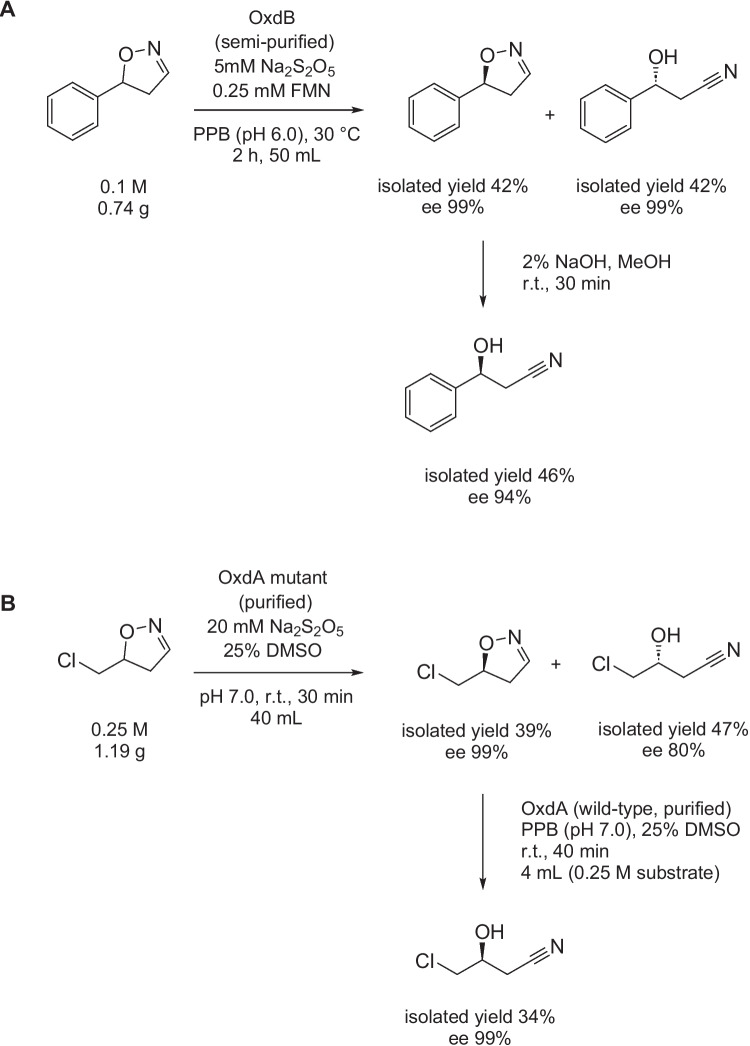
Synthesis of enantiopure β-hydroxynitriles from dihydroisoxazoles. **A** Synthesis of (*R*)- and (*S*)-3-hydroxy-3-phenylpropionitrile from (*R*,*S*)-5-phenyl-4,5-dihydroisoxazole (Zheng and Asano 2020). The first step was catalyzed by a semi-purified aldoxime dehydratase OxdB from *Bacillus* sp. (10 U/mL). **B** Synthesis of (*R*)- and (*S*)-4-chloro-3-hydroxybutanenitrile from (*R*,*S*)-5-(chloromethyl)-4,5-dihydroisoxazole (Zheng and Asano 2021). The first step was catalyzed by a purified aldoxime dehydratase OxdA-L318I (6.25 U/mL). This mutant exhibited an increased enantioselectivity for the substrate. The second step was catalyzed by the wild-type OxdA from *Pseudomonas chlororaphis* (62.5 U/mL). The nitrile yields were calculated per dihydroisoxazoles. PPB, potassium phosphate buffer; r.t., room temperature

Remarkably, also dihydroisoxazoles are Oxd substrates, as first shown for 1,2-benzisoxazole and 5-nitro-1,2-benzisoxazole, and undergo ring opening (a Kemp elimination reaction) in the active center of Oxds (Miao et al. 2017). This was used for the preparation of both enantiomers of synthetically useful β-hydroxynitriles such as 3-hydroxy-3-phenylpropionitrile (Fig. 7A). An asymmetric ring opening catalyzed by OxdB provided both the nitrile product and the unreacted dihydroisoxazole in excellent e.e. The unreacted substrate was converted to the corresponding nitrile under alkaline conditions in the subsequent step (Zheng and Asano 2020). An analogous approach was used for the synthesis of both enantiomers of 4-chloro-3-hydroxybutanenitrile (Fig. 7B) as precursors of l-carnitine and Atorvastatin (Zheng and Asano 2021). The dihydroisoxazoles were readily available starting from alkenes (Zheng and Asano 2020, 2021).

### In silico cascade design

As demonstrated in the previous chapters, Oxds can be integrated into enzymatic and chemoenzymatic multi-step reactions. Particularly the solvent tolerance of whole-cell catalysts harboring Oxds make them highly attractive for integration in chemoenzymatic routes where the chemical step requires non-aqueous or micro-aqueous conditions. For an efficient cascade design, databases such as the RetroBioCat database (available at retrobiocat.com) may be very useful: A target molecule can be dissected to precursor molecules and both biocatalytic and chemical functional group transformations are included. In frame of this review, we have curated data on enzymatic oxime dehydration from across the literature in the RetroBioCat database (Finnigan et al. 2023; RetroBioCat; A collection of tools for automated biocatalytic cascade design, Version: v2023.11.30, https://retrobiocat.com), allowing this information to be interactively explored and interrogated.

## Comparison of aldoxime dehydratase-catalyzed processes and other innovative approaches

Several of the processes mentioned above appear to be particularly promising for future transfer to the chemical industry. In particular, the syntheses of cinnamonitrile, citronellyl nitrile, substituted benzonitriles, nonanenitriles, or *n*-octanedinitrile (see above), as well as the syntheses of short chain aliphatic nitriles, phenylacetonitrile, 3-phenylpropionitrile, and heterocyclic nitriles described earlier (Xie et al. 2001; Kato et al. 1999) seem to be practicable mainly due to substrate concentrations that live up to industrial metrics (Table 3). In this section, we compare some of these enzymatic syntheses with alternative routes to the same nitriles to illustrate the advantages and disadvantages of the different approaches.

**Table 3:** Examples of nitriles synthesized with aldoxime dehydratases

Product	Substrate (mM)	Scale (L)	Conversion (%)	Isolated yield (%)	Reference(s)
Phenylacetonitrile	*Z*-Phenylacetaldoxime (500)	0.1	100	89	Xie et al. 2001
	Phenylacetic acid (10)	1	83	78	Horvat et al. 2022
3-Phenylpropionitrile	*Z*-3-Phenylpropionaldoxime (750)	0.1	99.5	90	Xie et al. 2001
*S*-2-Phenylpropionitrile	*E*-2-Phenylpropionaldoxime (50)	0.006	98.6	95.7	Chen et al. 2024
3-Hydroxy-3-phenylpropionitrile	5-Phenyl-4,5-dihydroisoxazole (100)	0.05	n.d	42 (*R*-nitrile); 46 (*S*-nitrile)	Zheng et al. 2020
*n*-Butyronitrile	*E/Z*-Butyraldoxime (100)	0.1	100	46	Xie et al. 2001
*n*-Valeronitrile	*n*-Valeraldoxime (250)	0.1	100	53	Xie et al. 2001
Isovaleronitrile	Isovaleraldoxime (200)	0.1	99.6	50	Xie et al. 2001
*n*-Hexanenitrile	*n*-Hexanaldoxime (300)	0.1	99.5	56	Xie et al. 2001
	1-Hexanol (1000)	≈ 0.03	≈ 89	60	Hinzmann et al. 2020a
*n*-Octanenitrile	*n*-Octanaldoxime (6982)	0.25	> 99	86	Hinzmann et al. 2019b
	1-Octanol (1000)	≈ 0.03	≈ 92	71	Hinzmann et al. 2020a
*n*-Octanedinitrile	*n*-Octandiol (500)	≈ 0.015	n.d.	61	Hinzmann et al. 2020a
*n*-Nonanenitrile	*n*-Octene (3823)	≈ 0.01	46	28	Plass et al. 2019
*n*-Decanenitrile	1-Decanol (1000)	≈ 0.03	≈ 90	63	Hinzmann et al. 2020a
*n*-Dodecanaldoxime	*n*-Dodecanenitrile (1000)	≈ 0.01	99	65	Yavuzer et al. 2023
*n*-Tetradecanaldoxime	*n*-Tetradecanenitrile (250)	≈ 0.01	> 99	89	Yavuzer et al. 2023
*n*-Hexadecanaldoxime	*n*-Hexadecanenitrile (250)	≈ 0.01	54	n.d	Yavuzer et al. 2023
Citronellyl nitrile	*E*,*Z*-Citronellal oxime (200)	≈ 0.05	> 99	n.d	Pei et al. 2023
*E*-Cinnamonitrile	*E*,*Z*-Cinnamaldoxime (100)	≈ 0.05	> 99	n.d	Pei et al. 2023
Benzonitrile, substituted benzonitriles	Benzaldehyde, substituted benzaldehydes (100)^a^	0.004–1	> 99	40–71	Zheng et al. 2022
	Benzylamine, substituted benzylamines (100)	≈ 0.01	40–76	30–69	Xiao et al. 2023
3-Cyanopyridine	*E*-Pyridine-3-carbaldehyde oxime (50)	0.3	n.d	98	Kato et al. 1999
2-Furonitrile	*E*-2-Furfurylaldoxime (100)	0.175	n.d	62	Kato et al. 1999

^a^Up to 700 mM for benzaldehyde in fed-batch mode

**Fig. 8:**
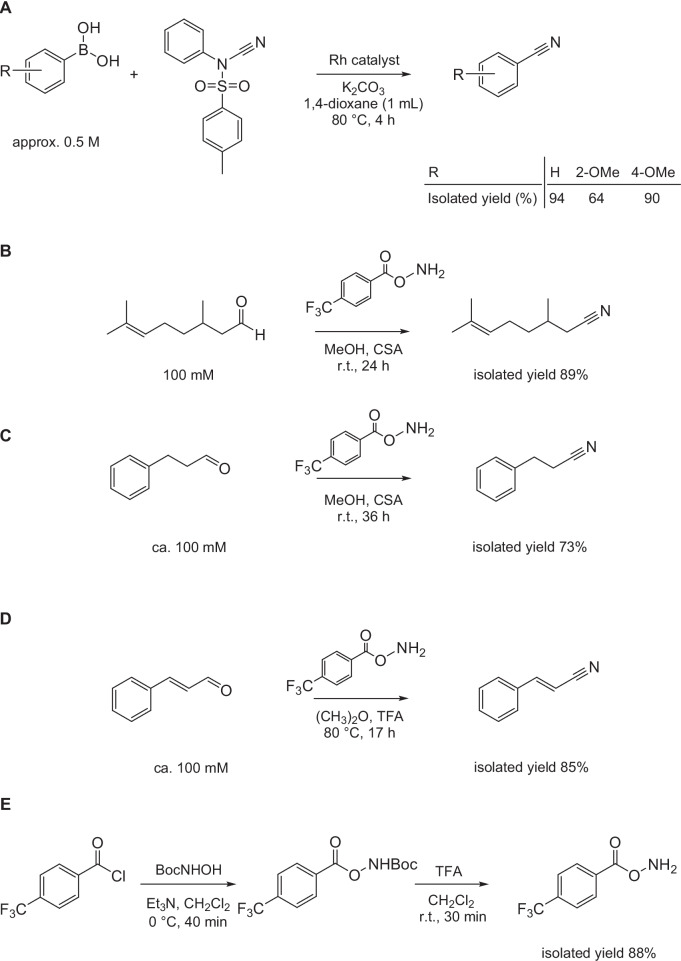
**A** Syntheses of **A** benzonitrile and substituted benzonitriles (Anbarasan et al. 2011), **B** citronellyl nitrile, **C** 3-phenylpropionitrile, and **D** cinnamonitrile (An and Yu 2015a). **E** Synthesis of *O*-(4-CF_3_-benzoyl)-hydroxylamine used in reactions (**B**–**D**) (An and Yu 2015b). CSA, L-(-)-camphorsulfonic acid; r.t., room temperature; TFA, trifluoroacetic acid

An innovation in nitrile synthesis (Anbarasan et al. 2011) is based on the cyanation approach where the conventional cyanation agents (see above) are replaced with the less toxic *N*-cyano-*N*-phenyl-*p*-toluenesulfonamide (NCTS) which is more eco-friendly and whose synthesis does not require a cyanide compound (Su et al. 2015; Li et al. 2016). The cyanation reaction using NCTS can be catalyzed by, e.g., a rhodium (III) complex, and the starting compounds are various boronic acids (Anbarasan et al. 2011; Soumya et al. 2021), heterocycles, or alkenes (Soumya et al. 2021) resulting in a vast number of potentially accessible nitriles. For example, boronic acids were converted to benzonitriles and substituted benzonitriles. The syntheses of products that were also prepared by the chemoenzymatic cascade explained above (Fig. 6B) are shown in (Fig. 8A). The boronic acids were used in higher concentrations than the amines, while the reaction time was longer, but the yields largely higher. Nevertheless, the chemical method requires a metal catalyst, a number of chemicals, and an elevated temperature.

Another innovative approach was used to produce citronellyl nitrile. The reaction starts from the corresponding aldehyde and uses *O*-(4-CF_3_-benzoyl)-hydroxylamine (CF_3_-BHA) as the nitrogen source and l-(-)-camphorsulfonic acid as the catalyst (Fig. 8B). An analogous route was applied to the synthesis of a number of other nitriles, including 3-phenylpropionitrile (Fig. 8C) and cinnamonitrile (Fig. 8D) (An and Yu 2015a). This innovative nitrile synthesis proceeds in one step with high yields, which is advantageous, but necessitates a prior two-step synthesis of CF_3_-BHA from 4-trifluoromethylbenzoyl chloride and *tert*-butyl *N*-hydroxycarbamate (An and Yu 2015b) (Fig. 8E). The enzymatic processes (Table 3) work with similar substrate concentrations and similar yields, but they are faster and use much less chemicals and organic solvents.

The critical point with the enzymatic processes is the cost of producing the catalyst. This factor cannot be currently assessed satisfactorily as a scale-up of Oxd catalyst production to more than a few liter scale is not yet realized. Nevertheless, it is justified to assume that Oxds can be produced at reasonable costs like many other industrially important enzymes.

## Conclusions

Nitrile-synthesizing enzymes clearly have an industrial potential, and, among them, Oxds are probably the most versatile. Particularly in the last 5 years, the spectrum of Oxds was significantly expanded, including Oxds with a different active site structure and diverging substrate specificities than the first Oxds discovered about 25 years ago. However, it is uncertain whether additional database searches will yield new Oxds that would significantly surpass the current ones. Although there are plethora of uncharacterized Oxds, these are also clusters of very similar enzymes. Moreover, evolutionarily distant Oxds often have similar substrate specificities. Therefore, a semi-rational design of mutants can be a more appropriate strategy and has already been applied to Oxds. Recently, the first functional immobilized Oxd were produced, which represents an additional strategy to improve the catalyst. Moreover, aldoxime dehydration by Oxds has been newly combined with a number of chemical steps, expanding the range of starting materials or allowing the nitrile products to be directly converted in cascade reactions. Some of the reactions are approaching a significant level of technological maturity. We speculate that scaling up the processes may lead to industrial applications in a number of cases. There have also been innovations in non-enzymatic nitrile synthesis, and some examples have been mentioned in this review. The decision on the choice of process must be based on a careful comparison of the advantages and disadvantages of each route.
